# MYC2b-regulated tyramine-derived phenolamides in maize defense against *Spodoptera frugiperda*

**DOI:** 10.3389/fpls.2026.1945110

**Published:** 2026-10-07

**Authors:** Yingying Zheng, Na Sun, Zicha Wu, Jinyi Tian, Jing Chen, Xueyang Ju, Rensen Zeng, Yuanyuan Song, Dongmei Chen, Jie Wang

**Affiliations:** 1 Key Laboratory of Ministry of Education for Genetics, Breeding and Multiple Utilization of Crops, College of Agriculture, Fujian Agriculture and Forestry University, Fuzhou, China; 2 State Key Laboratory of Agricultural and Forestry Biosecurity, College of Agriculture, Fujian Agriculture and Forestry University, Fuzhou, China; 3 Key Laboratory of Crop Biotechnology of Fujian Higher Education Institutes, College of Agriculture, Fujian Agriculture and Forestry University, Fuzhou, China; 4 Key Laboratory of Biological Breeding for Fujian and Taiwan Crops, College of Agriculture, Fujian Agriculture and Forestry University, Fuzhou, China; 5 Key Laboratory of Vector Biology and Pathogen Control of Zhejiang Province, School of Life Sciences, Huzhou Normal University, Huzhou, China

**Keywords:** MYC2, phenolamides, plant-herbivore interaction, *Spodoptera frugiperda*, tyramine hydroxycinnamoyl transferase

## Abstract

**Introduction:**

The fall armyworm *Spodoptera frugiperda* is a devastating pest of maize, capable of detoxifying the well-studied benzoxazinoid (BX) defenses. Whether maize deploys additional inducible defenses that complement BXs remains unclear.

**Methods:**

We performed targeted alkaloid metabolomics, absolute quantification, artificial-diet bioassays, transcript profiling, hormone quantification, and genetic analysis using a *myc2a myc2b* double mutant to investigate phenolamide-mediated defense against FAW in maize.

**Results:**

Upon *S. frugiperda* herbivory, maize leaves accumulated tyramine-derived phenolamides to levels that far exceeded those of BXs at 48 h after infestation. The two most abundant phenolamides, N-p- coumaroyltyramine and N-feruloyltyramine, reached over 50 mg/g fresh weight. N-feruloyltyramine significantly suppressed larval growth and survival, whereas N-p-coumaroyltyramine showed no significant effect; the methoxy group of the feruloyl side chain enhanced anti-insect activity. Phenolamide synthesis genes maintained high expression at 48 h, while BX biosynthetic genes were significantly downregulated at 24 h, accounting for the late-stage quantitative advantage of phenolamides. Herbivory activated jasmonate signaling. with MYC2b showing much stronger induction than MYC2a. The *myc2a myc2b* double mutant showed that both paralogs are required for phenolamide accumulation and tyramine hydroxycinnamoyl transferase (*THT*) expression. Given its stronger herbivory-responsive induction, MYC2b was selected for mechanistic verification.

**Discussion:**

Our findings establish that MYC2a/MYC2b-dependent tyramine-derived phenolamides, with MYC2b directly activating *THT3* transcription, constitute an important anti-herbivore specialized chemical defense.

## Highlight

Maize MYC2b directs the biosynthesis of tyramine-derived phenolamides to defend against fall armyworm.

## Introduction

Maize (*Zea mays*) is a staple crop worldwide, but its production is severely threatened by *Spodoptera frugiperda* (fall armyworm, FAW), a polyphagous lepidopteran pest that has rapidly spread across the globe (Ray et al., 2016; Day et al., 2017; Acevedo et al., 2018; Montezano et al., 2018; Sun et al., 2021; Jiang et al., 2022). Chemical pesticides remain the dominant control strategy, but their overuse threatens human health and drives pest resistance, posing major challenges to safe and efficient crop production (Wan et al., 2021; Zhang et al., 2021). Understanding the arsenal of maize defense mechanisms against this pest is critical for developing sustainable resistance strategies.

Benzoxazinoids (BXs), such as DIMBOA-;Glc and HDMBOA-;Glc, are among the most abundant defensive metabolites in maize and other cereals (Frey et al., 1997, 2009; Wouters et al., 2016). Upon herbivore attack, BX biosynthetic genes are induced within 12 hours, and the insecticidal compound HDMBOA-;Glc begins to accumulate locally at the feeding sites at approximately 24 hours post-;infestation (Maag et al., 2016). BXs exert potent anti-feedant and insecticidal activities against a range of chewing insects (Wouters et al., 2016), and their defensive functions have been extensively documented, establishing them as a core component of maize basal innate immunity. However, the rapid spread of FAW and its capacity to adapt to host defenses have broken this single-defense framework. Recent studies have confirmed that FAW has evolved efficient detoxification mechanisms to metabolize BXs. Key detoxification enzymes include UDP-;glycosyltransferases that glycosylate DIMBOA and HDMBOA (Glauser et al., 2011; Israni et al., 2020; Wang et al., 2024). This raises the possibility that maize may deploy additional, chemically divergent defense layers that function alongside or in succession to BXs to compensate for BX-mediated resistance after pest detoxification.

Phenolamides (also known as hydroxycinnamic acid amides) are jasmonate-;inducible specialized metabolites that have been implicated in plant defense against pathogens and insects across diverse plant species (Muroi et al., 2009; Bassard et al., 2010; Roumani et al., 2021; Zhou et al., 2023). In potato, tyramine-;derived phenolamides accumulate with delayed kinetics following wounding, with enzyme activity peaking at 36 hours and metabolite concentrations reaching significant levels only after several days (Negrel et al., 1993; Schmidt et al., 1999). In maize, mechanical wounding induces N-hydroxycinnamoyltyramines and tyramine hydroxycinnamoyltransferase (THT) activity (Ishihara et al., 2000). In addition, N-hydroxycinnamoyltyramines have also been reported to highly accumulated in maize plants infested by the generalist herbivore *Spodoptera littoralis* (Marti et al., 2013). In rice, jasmonate-activated *OsMYC2* has been demonstrated to drive phenolamide biosynthesis and enhance herbivore resistance, supporting a conserved defensive function of phenolamides in cereal crops (Liu M et al., 2025). Despite these observations, the quantitative contribution of phenolamides to direct defense against FAW in maize, particularly in relation to the well-; characterized BXs, remains largely unexplored. Moreover, the regulatory mechanism linking jasmonate signaling to phenolamide biosynthesis in maize; remains poorly understood. Specifically, whether the core jasmonate transcription factor MYC2 directly modulates this pathway has not been experimentally verified.

Phenolamide-mediated defense against insect herbivores has been well-documented in rice (*Oryza sativa*). For example, two major phenolamides, p-coumaroylputrescine and feruloylputrescine, are strongly induced by both chewing insectsand sucking insects, and dietary supplementation with these compounds at physiologically relevant concentrations significantly reduces insect survival (Alamgir et al., 2016). A broader survey identified four rice phenolamides—N-feruloylputrescine, N-feruloyltyramine, feruloylagmatine, and N1, N10-diferuloylspermidine—that are toxic to the white-backed planthopper *Sogatella furcifera* (Wang et al., 2020). Additionally, a recent study showed two tyramine-;derived phenolamides, N-;*p*-;coumaroyltyramine and N-;feruloyltyramine isolated from the weed *Humulus scandens* exerted insecticidal activity against FAW (Liu Y et al., 2025). While this work primarily screened these compounds as candidate botanical insecticides, the following critical questions remain unanswered in maize: (i) whether these compounds are endogenously produced as herbivory-inducible defensive metabolites upon herbivory; (ii) whether their accumulation is temporally coordinated with the known BX defenses; and (iii) how their biosynthesis is transcriptionally regulated in plants.

In this study, we performed targeted alkaloid metabolomics to simultaneously monitor BXs and phenolamides in maize leaves at 24 and 48 h post-infestation (h) after FAW infestation. We also examined the transcript levels of both BX and phenolamide biosynthetic genes. Using absolute metabolite quantification and artificial diet bioassays at 50 μg /mL, we validated the anti-herbivore activity of the two most abundant phenolamides. Through analysis of JA signaling, a *myc2* mutant, yeast one-hybrid, and dual-luciferase assays, we revealed that MYC2b-regulated tyramine-derived phenolamides are quantitatively dominant over BXs, sustained by persistent gene expression, and act as a chemically complementary defense, providing a potent second layer of protection against a highly adaptive pest. These results will strengthen our knowledge of specific phenolamides in maize to defend against herbivores, and provide theoretical basis for the development of plant-resistant varieties.

## Materials and methods

### Plant materials and growth conditions

Maize cultivar Zhengdan 958 was used throughout the study unless otherwise stated. The *myc2a myc2b* double mutant line was kindly provided by Dr. Lei Wang from Kunming Institute of Botany, Chinese Academy of Sciences. This mutant was generated from a KN5585 inbred line background using CRISPR/Cas9 technology (Ma et al., 2023). Maize seedlings were grown as previously described (Hu et al., 2022; Wang et al., 2022). Briefly, maize seeds were sown in premixed soil (PINDSTRUP, Denmark) to germinate. At the 1-2 leaf stage, seedlings were transplanted into small pots containing loam soil. All pots were maintained in a greenhouse (temperature: 28 ± 2°C, relative humidity: 70-75%, light: dark = 12 h:12 h), and plants cultured for 20-25 days (V3-V4) were used for all subsequent experiments.

### Insect rearing and herbivory treatment

FAW larvae were initially collected in Fuzhou City, Fujian Province, and reared in a growth chamber at 28 ± 2 °C with relative humidity of 70% ± 10% and a light: dark cycle of 14:10 h. Larvae were maintained on artificial diets composed of soybean powder, wheat bran, yeast powder and other ingredients as previously reported (Yao et al., 2024). Adults were provided with 10% honey solution, and a piece of paper towel was added for oviposition. For herbivory assays, we used maize seedlings at the V3-V4 developmental stage. For each plant, only the second uppermost fully expanded leaf was selected and enclosed in a single nylon mesh bag. One newly-molted third-instar FAW larva was placed into each bag. All treated plants were enclosed with nylon mesh to prevent insects from escaping, and larvae were allowed to feed for 24 h or 48 h before harvesting foliage samples. These time points were selected based on previous studies showing a strong induction of defenses at these time points (Glauser et al., 2011). Independent time-matched undamaged control plants were included for each sampling time-point (0, 24, and 48 h), and undamaged control plants received an identical nylon mesh bag on the same leaf position but without a larva. To enable simultaneous harvesting of all samples at the designated end-time, infestation treatments were staggered: plants assigned to the 48 h FAW infestation group were infested first, followed by plants for the 24 h feeding group, whereas 0 h control plants received no larval treatment. Undamaged control leaves were sampled from the identical leaf position. Leaf tissues were harvested surrounding insect feeding sites (approximately 1 cm radius around the damaged area), while tissues from undamaged plants were collected from equivalent leaf regions of the maize plants. All harvested leaf tissues were immediately frozen in liquid nitrogen (N_2_) and stored at -80 °C for further analyses.

### Alkaloids-targeted metabolomics analysis

Maize leaf samples collected after 24 h and 48 h of FAW infestation were lyophilized using a vacuum freeze dryer (Scientz-100F). The lyophilized tissues were then ground into fine powder using a ball mill (MM 400, Retsch) at 30 Hz for 1.5 min. Subsequently, 50 mg of powdered sample was weighed using an electronic balance (MS105DU), and 1200 μL of 70% methanolic aqueous solution (pre-cooled at −20 °C) supplemented with internal standard was added. Samples were vortexed for 30 s every 30 min, for a total of 6 vortex cycles. Following centrifugation at 12 000 rpm for 3 min, the supernatant was collected, filtered through a 0.22 μm microporous membrane and transferred into sample vials for subsequent UPLC-MS/MS analysis.

The analytical conditions are specified below as previously reported (Chen et al., 2013). UPLC separation was performed on an Agilent SB-C18 (1.8 µm, 2.1 mm × 100 mm); The mobile phase was composed of solvent A (pure water containing 0.1% formic acid), and solvent B (acetonitrile containing 0.1% formic acid). Gradient elution was carried out with the following program: the initial condition was set at 95% A, 5% B. A linear gradient to 5% A, 95% B was applied within 9 min, and this composition was kept for 1 min. Subsequently, the mobile phase was returned to 95% A, 5.0% B within 1.1 min and equilibrated for 2.9 min. The flow rate was set as 0.35 mL per minute; the column oven temperature was maintained at 40 °C; and the injection volume was 2 μL. The column eluate was directly coupled to an electrospray ionization (ESI) triple quadrupole-linear ion trap (QTRAP)-MS. The quantification of alkaloid-targeted metabolomics was performed by MetWare Co. Ltd. (Wuhan, China), and metabolites were identified using the MWDB database and quantified by multiple reaction monitoring. The metabolite data were analyzed via principal component analysis (PCA), orthogonal partial least squares discriminant analysis (OPLS-DA), cluster analysis and Pearson’s correlation analysis using the R software package MetaboAnalystR (Chong and Xia, 2018). For pairwise analysis, differential metabolites were identified based on variable importance in projection (VIP) value (VIP > 1) and absolute Log_2_ fold change (|Log_2_FC| ≥ 1.0). VIP values were extracted from OPLS-DA model result, which also included score plots and permutation plots. The data was log-transformed (log_2_) and mean centering before OPLS-DA. To prevent model overfitting, a permutation test with 200 iterations was also performed.

### Analysis of phenolamide and DIMBOA contents in maize plants

The absolute contents of N-;*p*-;coumaroyltyramine, N-;feruloyltyramine and DIMBOA in maize leaves were measured as previously described with slight modifications (Zhuang et al., 2022). Briefly, 100 mg fresh weight (FW) of individual leaf samples collected from maize plants infested with FAW were ground into a fine powder and then 1 mL of 70% methanol was added. After vortexing for 5 min, samples were sonicated in an ultrasonic bath for 20 min and then centrifuged at 12000 rpm for 10 min at 4 °C. The supernatant of each sample was collected and filtered using a microporous membrane (0.22 μm pore size, Millipore). Methanol in the filtrate was evaporated under a gentle stream of nitrogen, and the remaining aqueous fraction was lyophilized with a vacuum freeze dryer. The extract was reconstituted in 150 μL of 70% methanol and analyzed by liquid chromatography–tandem mass spectrometry (LC-MS/MS) with an ESI triple quadrupole-linear ion trap mass spectrometer (Waters, USA). Commercially available authentic standards of N-p-coumaroyltyramine (CAS: 36417-86-4), N-feruloyltyramine (CAS: 66648-43-9) and DIMBOA (CAS: 15893-52-4) were purchased from Beijing Psaitong Biotechnology Co., Ltd. (Beijing, China) and used as external calibrants. The extraction efficiency was assessed by spiking known amounts of authentic standards into leaf samples prior to extraction, and the recovery rates for N-p-coumaroyltyramine and N-feruloyltyramine were determined to be 82% and 86%, respectively. Metabolite concentrations were quantified using external standard curves; no internal standard was added to the samples. All treatments were performed with three independent biological replicates.

### Determination of direct effects of phenolamides on the performance of FAW larvae

To determine the growth-inhibitory effects of tyramine-derived phenolamides on FAW larvae, commercial analytical standards of N-;*p*-;coumaroyltyramine and N-;feruloyltyramine were supplemented into artificial diets to evaluate their direct anti-insect activity. Both compounds were dissolved in methanol and mixed thoroughly into artificial diets at a final concentration of 50 μg/mL as described by Liu Y et al. (2025). Each 2-day-old post-hatching larva was placed individually into a 22.5 mL plastic cup containing a diet cube (c.a. 1 g) spiked with N-;*p*-;coumaroyltyramine or N-;feruloyltyramine. Each treatment included 30 independent biological replicates. Larval weight gain and mortality were recorded after 5 days of continuous feeding. To further explore the structure–activity relationship of the two phenolamides, the effects of their precursor compounds *p*-coumaric acid and ferulic acid on larval growth were also assessed. As reported in a previous study (Shi et al., 2024), both *p*-coumaric acid and ferulic acid were dissolved in acetone and added to artificial diets at final concentrations of 5 mg/mL and 10 mg/mL for each compound. Stock solutions of N-*p*-coumaroyltyramine and N-feruloyltyramine were prepared in methanol to reach a final methanol concentration of 0.1% (v/v) in artificial diet, and diet with 0.1% (v/v) methanol served as vehicle control. For *p*-coumaric acid and ferulic acid treatments, stock solutions were prepared in acetone, giving a final acetone concentration of 0.2% (v/v) in diet, and diet containing 0.2% (v/v) acetone was included as matched vehicle control. Each treatment consisted of 30 2-day-old newly-hatching larvae, and weight gains of surviving larvae were recorded after 5 days. Notably, newly-hatched 2-day-old FAW larvae show intrinsic background mortality under individual rearing in small plastic cups. Diet deterioration and occasional mold formation were closely monitored during the bioassay, and larvae from cups with visible mold were excluded from further analysis.

### Quantification of jasmonic acid and JA-isoleucine

Samples collected from maize plants infested by FAW larvae for 24 h and 48 h were used to quantify endogenous concentrations of JA and JA-Ile. The quantification and analysis processes were conducted as previously described (Wu et al., 2007). Briefly, the maize leaves were harvested for the analyses and 3 leaves of the same treatment were pooled as one sample. Three independent biological replicates were analyzed for each treatment at each time point. The frozen maize leaves were ground in liquid nitrogen, and 150 mg of each sample was stored in dry ice and shipped for quantification at the Plant Hormone Platform of Kunming Institute of Botany, Chinese Academy of Sciences.

### Effect of MeJA treatment on the biosynthesis of phenolamide

To determine the effects of JA on biosynthesis of tyramine-derived phenolamides in maize, the maize seedlings were exposed to exogenous methyl jasmonate (MeJA), a known elicitor of plant anti-herbivore defense response, by spraying with 100 μM of MeJA dissolved in 0.1% acetone. Control plants were sprayed with an equal volume of 0.1% acetone solution as a mock treatment. Leaf samples were harvested 24 h and 48 h after treatment with MeJA and frozen in liquid N_2_, and stored in −80 °C until subsequent analysis of tyramine N-hydroxycinnamoyl transferase (THT) transcript levels and concentrations of N-;*p*-;coumaroyltyramine or N-feruloyltyramine.

### RNA extraction, cDNA synthesis, and quantitative real-time PCR analysis

Frozen maize leaf samples collected from each treatment were ground to fine powder in liquid N_2_ with a mortar and pestle, and subsequently total RNA was extracted using TRIzol Reagent. First-strand cDNA was synthesized using HiScript III All-in-One RT SuperMix. The transcript levels of all target genes involved in the JA signaling pathway and phenolamide biosynthesis were quantified by quantitative real-;time PCR (qRT-;PCR) using Taq Pro Universal SYBR qPCR Master Mix. *ZmActin* was employed as the internal reference gene, and relative gene expression was calculated using the 2^-;ΔΔCt^ method (Livak and Schmittgen, 2001). Each treatment included three independent biological replicates, and each biological replicate was measured with two technical replicates. Primers were designed using Primer 6 software, and listed in **Supplementary Table S1**.

### Yeast one-hybridand dual-luciferase reporter assay

Promoter fragments of THT genes harboring putative MYC2 binding sites and the full-;length coding sequence (CDS) of MYC2 were cloned into pAbAi (bait) and pGADT7 (prey) vectors, respectively. Protein-;DNA interactions were verified based on yeast growth on synthetic dropout SD/-;Ura/-;Leu medium supplemented with aureobasidin A (AbA). For dual-;luciferase reporter assays, the *THT3* promoter and MYC2 CDS were cloned into pGreenII-0800luc (reporter) and pGreenII-62SK (effector) vectors, respectively. The recombinant plasmids were co-transformed into *Nicotiana benthamiana* leaves via *Agrobacterium*-mediated transient transformation, and relative luciferase activity was detected using a Dual-Luciferase Reporter Assay System. All primer sequences used for vector construction are listed in **Supplementary Table S1**.

### Statistical analysis

Statistical analysis was performed using SPSS Statistics software (Version 19.0). The data were subjected to Student’s *t*-test or analysis of variance (ANOVA) followed by Tukey’s post-;hoc test following the Shapiro–Wilk normality test, and differences with a *P* value < 0.05 was considered statistically significant between treatments. Larval survival curves were compared using the log-rank (Mantel-Cox) test, with the significance threshold set at *P* < 0.05. All values are presented as mean ± standard error (SE), and figures were generated using GraphPad Prism (Version 8.0).

## Results

### Targeted metabolomics reveals massive accumulation of tyramine-derived phenolamides

The accumulation of BXs in maize during herbivore infestation has been widely reported, while the accumulation profiles of phenolamides in maize infested by FAW has not been well-explored. To characterize the temporal dynamics of these defensive metabolites upon FAW attack, we performed targeted alkaloid metabolomics on maize leaves, with leaf samples harvested at 24 and 48 h post-;infestation as shown in **Figure 1A**. The metabolomics data showed high reproducibility according to Principal component analysis (PCA) score plot and overlay of total ion current (TIC) chromatograms of quality control (QC) samples (**Supplementary Figure S1**), and we detected a total of 154 alkaloid-specific metabolites (**Supplementary Table S2**). PCA revealed a clear separation among the control (CK), FAW_24, and FAW_48 groups, thus confirming significant metabolic divergence induced by FAW infestation (**Figure 1B**). Volcano plot analysis identified 154 differential accumulated metabolites (DAMs) in both treatment groups: Specifically, 34 DAMs were upregulated and 7 DAMs were downregulated at 24 h post-;infestation (**Figure 1C**), while 61 DAMs were upregulated and 6 downregulated at 48 h post-;infestation (**Figure 1D**). Consistent with the temporal dynamics of defense induction, more DAMs were detected at 48 h post-;FAW infestation than at 24 h. The DAMs identified at 48 h post-;infestation (**Supplementary Table S3**) were selected for in-;depth characterization. Hierarchical clustering analysis showed that alkaloids (34.67%, 26 metabolites) and phenolamides (32%, 24 metabolites) were among the most prevalent groups through hierarchical clustering analysis (**Figure 1E**). Specifically, eight differentially accumulated BXs were detected in the clustered class of alkaloids, the contents of 6 out of 8 BXs were found to increase more than 2-fold compared to uninfested controls, including three N-methoxylated benzoxazinone derivatives (HDMBOA, HDMBOA-glc, and HBOA-glc), MBOA, DIBOA and D-DIBOA. In contrast, we also observed that two BXs DIMBOA and HBOA were significantly declined in maize plants FAW-;infested (**Figure 1F**). For the cluster of phenolamides, the tyramine-derived phenolamides (50%, 12 metabolites) constituted the predominant subclass followed by the group of putrescine-derived phenolamides (25%, 6 metabolites) (**Figure 1G**). Among these differentially accumulated tyramine-derived phenolamides, the relative abundance of coumaroyltyramine derivatives highly accumulated over 1000-fold in FAW-;infested maize plants relative to controls, followed by feruloyltyramine derivatives with an approximately 250-;fold increase (**Figure 1H**). Collectively, these results demonstrate that maize responds to FAW herbivory with a late, massive accumulation of tyramine-derived phenolamides that quantitatively dominate the induced metabolome at 48 h post-infestation.

**Figure 1:**
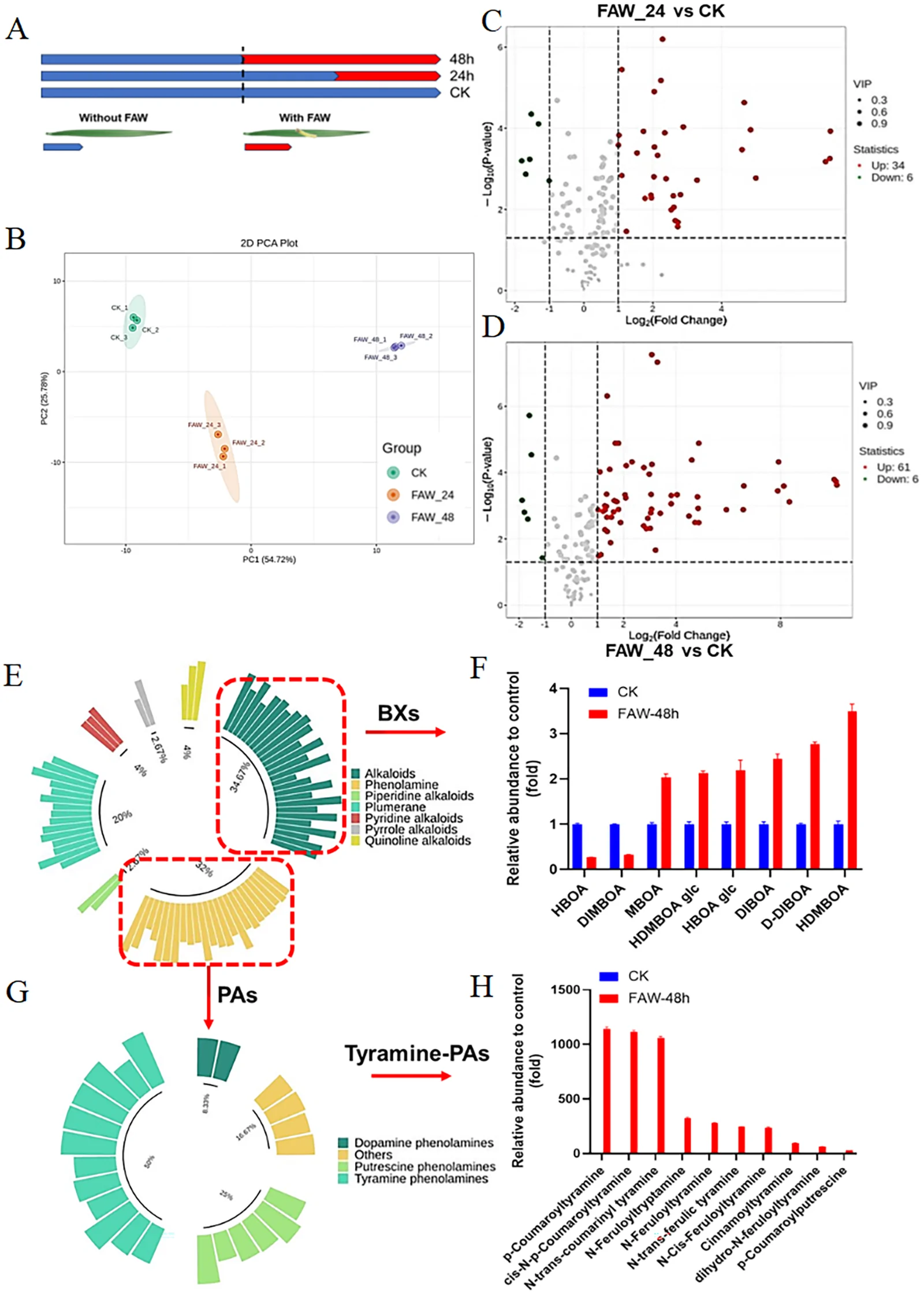
Targeted metabolomics reveals massive accumulation of tyramine-derived phenolamides. **(A)** Design of the FAW feeding experiments. The second uppermost fully expanded leaf from V3-4 maize seedling was enclosed in a nylon mesh bag. One newly-molted 3^rd^ instar FAW larva was added to each bag, and leaf tissue was harvested after 0-48 h of FAW feeding. **(B)** Principal component analysis (PCA) plot of all detected metabolites in maize plants by metabolome profiling. Data points indicate the respective time points as feeding progressed. Volcano plots demonstrating numbers of differentially accumulated metabolites (DAMs) that are upregulated and downregulated in maize plants damaged by FAW at 24 h post-infestation **(C)** or 48 h post-infestation **(D)** compared to undamaged control plants. Color coding represents the intensity of induction (red) with Log_2_ (Fold-change)≥1or suppression (blue) with Log_2_ (Fold-change)≤-1 of metabolites. **(E)** Proportional distribution of different alkaloid classes in maize leaves after FAW feeding. **(F)** Relative abundance of differentially accumulated BXs in maize leaves after FAW feeding. **(G)** Proportional distribution of different phenolamides in maize leaves after FAW feeding. **(H)** Relative abundance of differentially accumulated tyramine-derived phenolamides in maize leaves after FAW feeding. Fold values have been calculated from the relative mean peak area of each compound and normalized to control levels (n=3), and significance was analyzed using Student’s *t*-test (*P* < 0.05).

### Transcriptional analysis reveals temporal complementarity: phenolamide biosynthetic genes remain highly expressed whereas BX genes decline

To understand the transcriptional basis of the metabolic shift, we examined the expression of BX biosynthetic genes (*Bx1*, *Bx9*, *Bx10*) and phenolamide biosynthetic genes (*PAL*, *4CL*, *TDC*, *THT3*) at 0, 24, and 48 h via qRT-;PCR. *Bx1*, *Bx9*, and *Bx10* were significantly induced at 24 h but declined markedly by 48 h (**Figure 2A**). In contrast, *PAL*, *4CL*, *TDC*, and *THT3* showed sustained or even increased expression at 48 h (**Figure 2B**). Notably, *THT3* exhibited the strongest induction, with a 10-;fold at 48 h. These expression patterns, which show early transient activation of BX genes followed by late sustained activation of phenolamide biosynthetic genes, provide strong transcriptional evidence for temporal complementarity between the two defense pathways.

**Figure 2:**
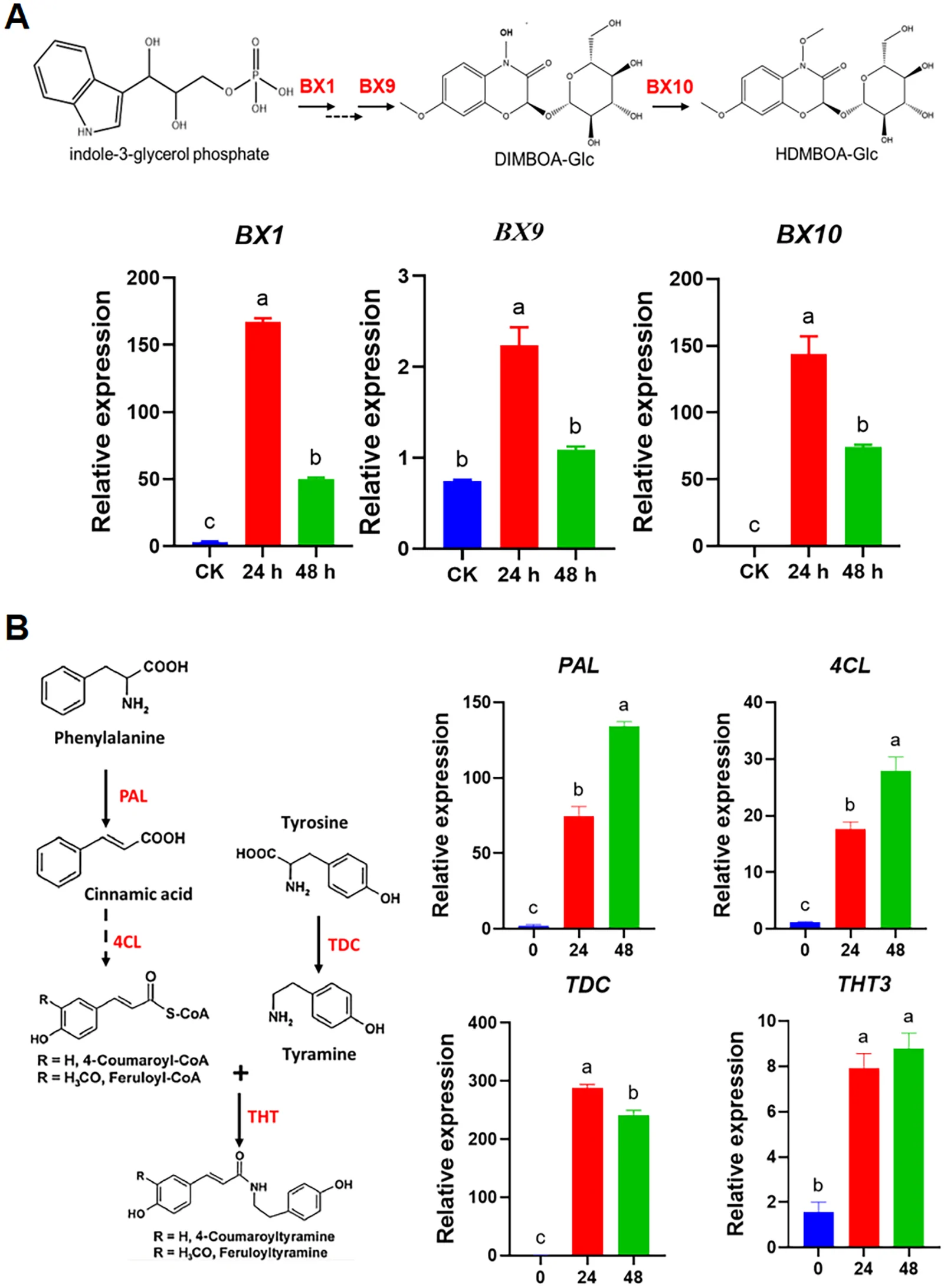
Transcriptional analysis of both BXs and PAs biosynthetic genes. **(A)** Expression of BX biosynthetic genes (*Bx1*, *Bx9*, *Bx10*) at 0, 24, and 48 h after FAW infestation. **(B)** Expression of PA biosynthetic genes (*PAL*, *4CL*, *TDC*, *THT3*) at 0, 24, and 48 h after FAW infestation. Statistical significance was analyzed using Tukey’s test in a one-way ANOVA (*P* < 0.05, n = 3), with different letters indicating differences between treatments.

### Tyramine-derived phenolamides directly suppress FAW larval growth and survival, and the methoxy group enhances bioactivity

In order to investigate the functions of these inducible tyramine-derived phenolamides in maize defense against herbivores, N-;*p*-;coumaroyltyramine and N-;feruloyltyramine were selected to evaluate their effects on the performance of FAW. Absolute quantification with analytical standards revealed that the two tyramine-;derived phenolamides accumulated in maize plants infested by FAW compared to uninfested controls (**Figure 3A**). Basal concentrations of N-;*p*-;coumaroyltyramine and N-;feruloyltyramine in undamaged control leaves were extremely low, near the detection limit of our LC-MS/MS assay, and substantial accumulation was only triggered by FAW herbivory. For N-;*p*-;coumaroyltyramine content, 53.57 µg/g and 19.53 µg/g were detected in maize plants infested by FAW for 24 h and 48 h, respectively. For N-;feruloyltyramine content, 69.65 µg/g and 50.29 µg/g were detected in maize plants infested by FAW for 24 h and 48 h, respectively. These data indicate that tyramine-derived phenolamides, especially the N-;feruloyltyramine, still accumulated in maize plants during late FAW infestation. In contrast, the absolute concentrations of DIMBOA, were significantly lower, at approximately 2.5 μg/g FW at 24 h and 1.5 μg/g FW at 48 h (**Supplementary Figure S2**). Thus, the combined absolute content of N-;*p*-;coumaroyltyramine and N-;feruloyltyraminewas over 10-;fold higher than that of DIMBOA at 48 h. To functionally validate their anti-;herbivore activity, artificial diets supplemented with 50 µg/mL N-;*p*-;coumaroyltyramine or N-;feruloyltyramine were used assess their effects on FAW larval growth. For reference, 50 µg/mL corresponds to approximately 148 μM for N-feruloyltyramine and 164 μM for N-*p*-coumaroyltyramine. Note that simple direct conversion between leaf tissue concentrations (μg/g FW) and artificial diet concentrations (µg/mL) cannot be achieved, as gut-associated dilution and compound metabolism alter the real compound exposure experienced by larvae. The 50 µg/mL dose was chosen to reflect the upper molar range of phenolamide levels detected in FAW-infested maize leaves.The larval weight gains were significantly inhibited in FAW fed on artificial diets containing 50 µg/mL N-;feruloyltyramine compared to the controls, while 50 µg/mL N-;*p*-;coumaroyltyramine showed no significant effect on larval growth over a 5-day period (**Figures 3B, C**). Survival analysis over 5 days revealed that N-feruloyltyramine significantly reduced larval survival (log-rank test, *P* < 0.001). By day 5, the mortality rate in the N-feruloyltyramine group reached 60%, compared with 30% in the control group and 40% in the N-p-coumaroyltyramine group (**Figure 3D**). To explore the structural basis for the differential activity between the two phenolamides, we tested their respective cinnamoyl precursors—*p*-coumaric acid and ferulic acid—in larval feeding assays at 5 mg/mL and 10 mg/mL. The chemical structures of *p*-coumaric acid and ferulic acid are shown in **Figure 3E**, and there is an additional methoxy group at C-3’ in ferulic acid. The bioassay showed that ferulic acid caused significantly greater growth inhibition than *p*-;coumaric acid over a 5-;day period (**Figure 3F**). Collectively, these results demonstrated that tyramine-derived phenolamides from infested maize leaves function as potent anti-herbivore metabolites against FAW. The methoxy group on the feruloyl moiety strongly contributes to this anti-herbivore activity, providing a structural basis for the higher bioactivity of N-feruloyltyramine compared with N-*p*-coumaroyltyramine.

**Figure 3:**
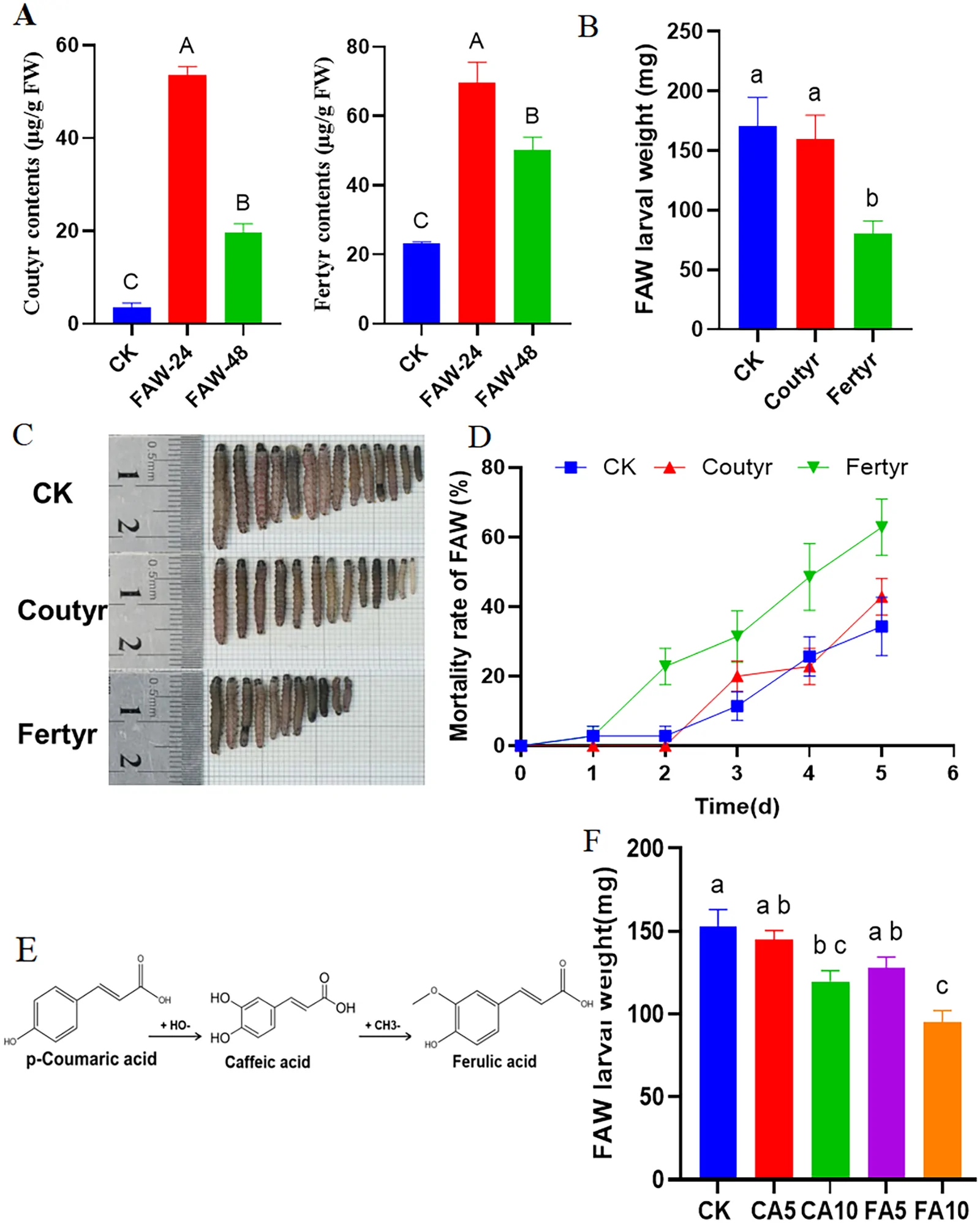
Tyramine-derived phenolamides directly suppress FAW larval growth and survival, and the methoxy group enhances bioactivity. **(A)** Absolute quantifications of N-*p*-coumaroyltyramine (Coutyr) and N-feruloyltyramine (Fertyr) in maize leaves infested by FAW for 24 h and 48 h. **(B)** Average larval weight gain `of FAW larvae feeding on diet containing 50 µg/mL of Coutyr and Fertyr. Data are presented as mean ± SE (n=30), and different letters indicate significant differences (one-way ANOVA, Tukey’s HSD, *P* < 0.05). **(C)** Growth phenotype of insects fed on artificial diet supplemented with Coutyr and Fertyr for 5 days. **(D)** Kaplan-Meier survival curves of larvae treated with Coutyr and Fertyr during 5 days (log-rank test, *P* < 0.05). **(E)** Two-step conversion of p-coumaric acid to ferulic acid via caffeic acid as the intermediate. **(F)** Larval weight at day 5 after feeding on *p*-coumaric acid or ferulic acid. Larval weight at day 5 after feeding on p-coumaric acid (CA) or ferulic acid (FA) at 5 mg/mL (CA5, FA5) and 10 mg/mL (CA10, FA10). Data are presented as mean ± SE (n=30), and different letters indicate significant differences (one-way ANOVA, Tukey’s HSD, *P* < 0.05).

### Herbivory activates jasmonate signaling and MYC2 expression

To investigate the upstream signaling controlling phenolamide biosynthesis, we measured the levels of JA and its bioactive conjugate JA-Ile in leaves at 0, 24, and 48 h. Both JA and JA-;Ile increased significantly at 24 h, while still remained elevated at 48 h compared to the controls (**Figure 4A**). qRT-PCR analysis showed that the JA biosynthetic genes *LOX1* and *AOS* were significantly induced at both 24 and 48 h (**Figure 4B**). Notably, the transcript levels of both *MYC2a* and *MYC2b* increased dramatically at 24 h and remained high at 48 h, with *MYC2b* exhibiting a particularly strong response to FAW herbivory (**Figure 4C**). Consistent with these findings, exogenous MeJA treatment significantly induced *THT3* expression and increased the accumulation of Coutyr and Fertyr (**Supplementary Figure S3**). Collectively, these results demonstrate that FAW herbivory activates the JA signaling cascade, including the core transcription factor MYC2, which forms the upstream regulatory framework governing phenolamide biosynthesis in maize.

**Figure 4:**
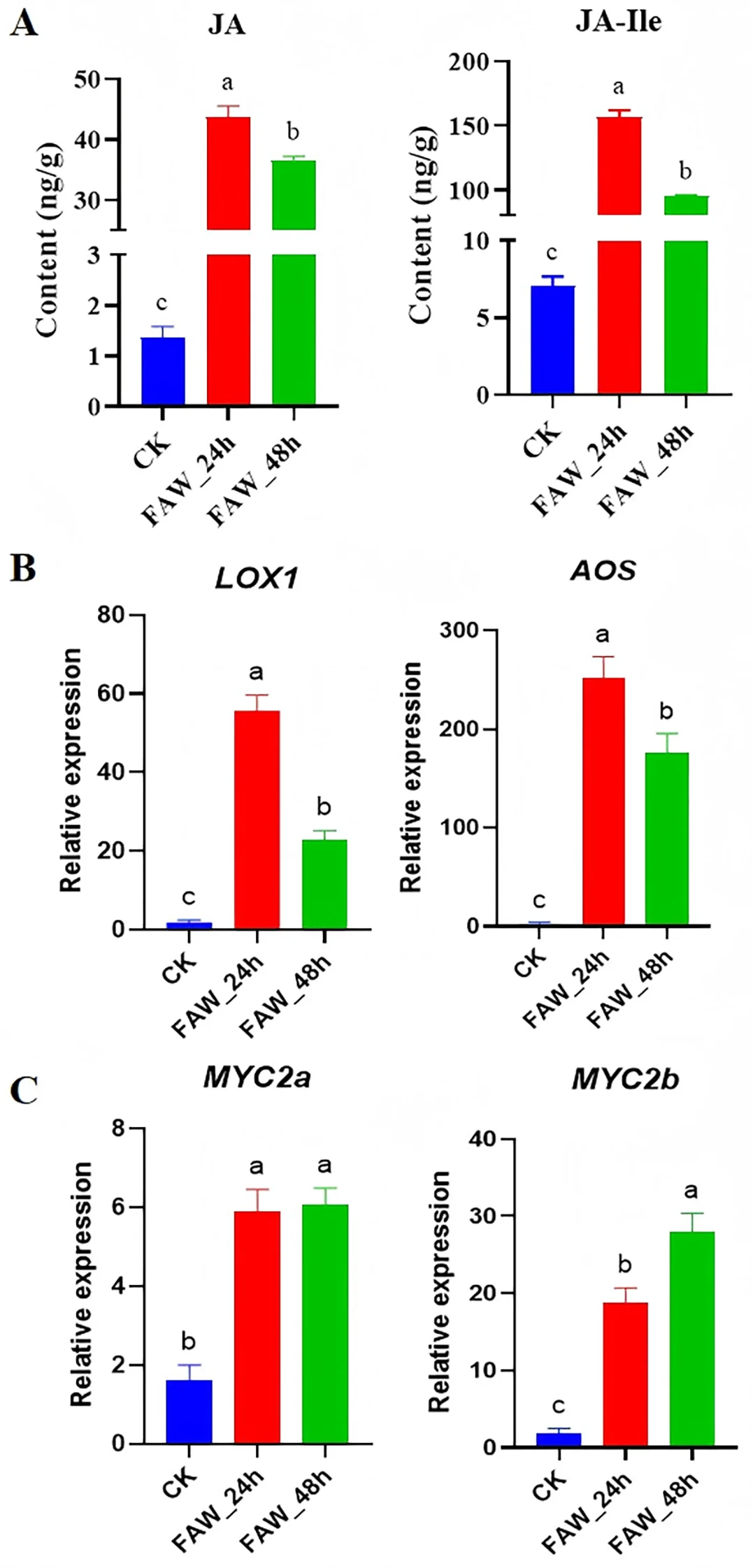
Herbivory activates jasmonate signaling and MYC2 expression. **(A)** Contents of jasmonic acid (JA) and JA-Ile in maize leaves at 0, 24, and 48 h. **(B)** Transcript levels of JA biosynthetic genes *LOX1* and *AOS*. **(C)** Transcript levels of JA signaling-;related transcription factor genes *MYC2a* and *MYC2b*. All gene expression samples were collected at 0, 24, and 48 h post-FAW infestation. Data are presented as mean ± SE, and different letters indicate significant differences (one-way ANOVA, Tukey’s HSD, *P* < 0.05).

### MYC2 directly regulates the phenolamide pathway by binding to the *THT3* promoter

To test whether MYC2 is required for phenolamide accumulation, we used a JA signaling-deficient *myc2a myc2b* double mutant (hereafter designated *myc2*) to validate the role of JA signaling in regulating the biosynthesis of tyramine-derived phenolamides. Concentrations of both Coutyr and Fertyr were significantly decreased in *myc2* mutant plants compared with wild-;type (WT) plants (**Figure 5A**). The expression of all three *THT* genes in mutant maize plants was significantly lower than that in WT plants with FAW infestation, whereas *THT2* and *THT3* showed no significant difference in FAW-infested mutant plants compared to those mutants without infestation (**Figure 5B**). Previous work using this identical genetic material demonstrated that JA and JA-Ile accumulate to similar levels in WT and myc2a myc2b double mutant plants under herbivory-mimic treatment (Ma et al., 2023). This observation is consistent with MYC2 functioning downstream of JA-perception; reduced phenolamide accumulation in the double mutant is therefore attributable to impaired transcriptional activation of biosynthetic genes, rather than diminished JA/JA-Ile hormone pools. The homologous genes of *THT* gene cluster components found in maize were displayed in **Figure 5C**, and *THT1* was distributed distantly from *THT2* and *THT3*, which are clustered nearby. Based on the much stronger induction of *MYC2b* compared to *MYC2a* upon FAW herbivory (**Figure 4C**), we focused on *MYC2b* for further mechanistic analysis. Y1H assays confirmed direct binding of *MYC2b* to 2kb-promoter regions of *THT*s. Putative MYC2 binding motifs were predicted using the STREME algorithm with default parameters (**Supplementary Figure S4**). The promoters of *THT*s contained 2 to 5 putative MYC2-binding motifs, among which there were five binding motifs in *THT3* (**Figure 5D**). Following AbA screening to determine the optimal selection concentration, the bait strain carrying the T3P4 fragment of the *THT3* promoter showed specific growth on medium supplemented with 300 ng/mL AbA when co-expressed with *MYC2b*, confirming the direct interaction between *MYC2b* and the T3P4 region (**Figure 5E**). Then a transient expression assay was performed to test whether *MYC2b* regulated the expression of *THT*s using the dual-luciferase reporter system. LUC driven by the *THT3* promoter and REN driven by the CaMV 35S promoter as an internal control, together with an effector plasmid expressing *MYC2b* were verified and expressed in the protoplasts of tobacco seedlings. We found that co-expression of *MYC2b* with LUC driven by the *THT3* promoter significantly increased the LUC/REN ratio, but not in the empty vector control (**Figure 5F**). These results confirm that *MYC2b* directly binds to the *THT3* promoter and activates its transcription, establishing a direct JA-*MYC2b*-THT regulatory module for phenolamide biosynthesis in maize. It should be noted that the double-;mutant phenotype reflects the collective in-;vivo requirement of both MYC2a and MYC2b for phenolamide accumulation under FAW herbivory. Our heterologous Y1H and dual-;luciferase assays only characterized MYC2b, demonstrating its capacity to bind and activate the *THT3* promoter. We cannot rule out the possibility that MYC2a also binds and regulates *THT* gene promoters. Further experiments, including heterologous assays for MYC2a and phenotypic analysis of single-;mutant lines, are needed to resolve the paralog-;specific functions of MYC2a and MYC2b.

**Figure 5:**
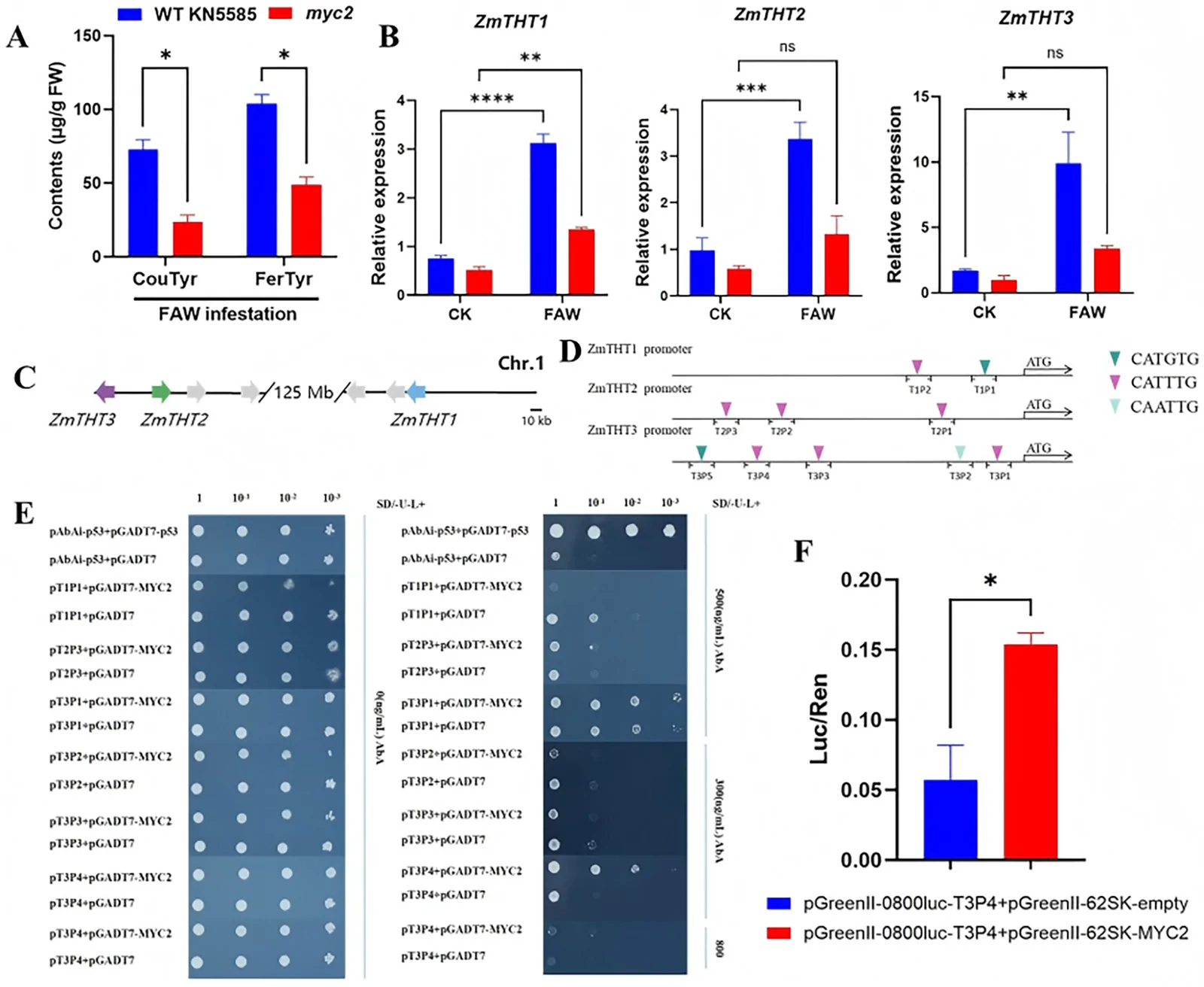
MYC2b regulates the phenolamide pathway by binding to the THT3 promoter. **(A)** The content of Fertyr and Coutyr in *myc2* mutant and WT plants after FAW infestation. **(B)** Transcript levels of the biosynthesis-related genes (*THT1/2/3*) of tyramine-derived phenolamides in *myc2* mutant and WT plants after FAW infestation. **(C)** Graphical map of the homologous genes of HT gene cluster components found in *Zea may*. Colored arrows: gene cluster components; gray arrows: other genes. **(D)** Schematic of the 2-kb length promoter of *THT*, and triangles indicate potential binding sites in the promoter sequence by the transcription factor MYC2. **(E)** Screening assays of *MYC2b* interacting fragments. **(F)** *MYC2b* activates the transcription of *THT3*. *N. benthamiana* leaves were infiltrated with different combinations of effectors and reporters. The Luc activity was normalized to the REN activity as an internal control. Ren, Renilla luciferase; Luc, firefly luciferase. Data are presented as mean ± SE (n=3), and statistical significance was analyzed using Student’s t-test (*P < 0.05; **P < 0.01; ***P < 0.001; ****P < 0.0001).

## Discussion

A core finding of this work is that maize activates two distinct classes of specialized defensive metabolites: benzoxazinoids (BXs) and phenolamides, which exhibit divergent accumulation dynamics and regulatory patterns. Absolute metabolite quantification demonstrated that tyramine-based phenolamides outaccumulate major BXs as early as 24 h after herbivory, with this disparity further amplified at 48 h post-infestation. Accordingly, phenolamides constitute the quantitatively primary defensive metabolites across the 24–48 h feeding period, whereas BXs function as a secondary yet biologically relevant defensive component. Several factors may account for this quantitative difference. For example, the developmental stage of maize plants used in this study (V3–V4) may alter the relative contribution of distinct specialized defense pathways. Young maize seedlings accumulate substantially higher BX concentrations than older plants, and BX biosynthetic genes exhibit clear tissue-specific expression patterns in early vegetative tissues (Zhou et al., 2018). In contrast, phenolamide pathways exhibit greater basal activity in late vegetative stages and display stronger metabolic induction under biotic stress (Marti et al., 2013). In addition, we found BX biosynthetic genes (*Bx1*, *Bx9*, *Bx10*) were strongly induced at 24 h, but declined markedly by 48 h, which is consistent with a previous report showing that BX biosynthetic genes and BX contents were induced within 24 h and afterwards (Maag et al., 2016). In contrast, phenolamide biosynthetic genes (*PAL*, *4CL*, *TDC*, *THT3*) remain highly expressed or even increase at 48 h, thereby sustaining the production of phenolamides. This divergence in expression kinetics—transient induction for BX genes versus sustained for phenolamide genes—explains why phenolamides continue to accumulate and maintain their quantitative dominance at later time points, while BX accumulation plateaus or declines. These patterns can be interpreted within the framework of cost-effective defense deployment (Wan et al., 2025). Herbivory triggers fast but temporary BX induction to limit metabolic overconsumption (Frey et al., 2000). As phenylpropanoid-derived metabolites, phenolamides function in both direct insect resistance and cell wall reinforcement (Bassard et al., 2010), and are maintained at higher basal and induced levels, providing lasting defense. Maize employs a tiered defensive scheme: transient BX bursts offer instant protection, while stable phenolamide accumulation sustains robust resistance against FAW feeding. In the present study, metabolite profiling was restricted to 0 h, 24 h and 48 h post-;infestation; we lack metabolite measurements for later time-;points (>48 h). Although phenolamide biosynthetic genes maintain high expression at 48 h, whether phenolamide concentrations remain high or decline after 48 h in infested maize leaves remains uncharacterized. Future sampling extending beyond 48 h will help clarify the long-term persistence of these defensive metabolites in planta.

Beyond their quantitative difference, BXs and PAs differ fundamentally in their modes of action against insect herbivores. BXs, particularly DIMBOA and its glucosylated forms, function as chemical toxins that disrupt insect digestive physiology. Upon ingestion, DIMBOA-Glc is hydrolyzed by insect β-glucosidases to the active aglycone DIMBOA, which interferes with digestive proteases and nutrient assimilation, leading to reduced growth and increased mortality (Wouters et al., 2016; Zhou et al., 2018). However, FAW has evolved effective counter-defenses: UDP-glycosyltransferases (UGTs) can re-glycosylate DIMBOA and HDMBOA, rendering them less toxic (Israni et al., 2020; Wang et al., 2024). This detoxification capacity is a key factor limiting BX efficacy against this pest.

Phenolamides, in contrast, are structurally distinct and likely exert their effects through different mechanisms. Several studies have demonstrated that phenolamides can cross-link with cell wall components, particularly through oxidative coupling to polysaccharides and lignin, thereby reinforcing physical barriers and reducing nutrient accessibility for herbivores (Macoy et al., 2015; Liu et al., 2022). This cell wall-fortifying function represents a mechanism distinct from the direct toxicity of BXs and may provide a more durable defense that is not easily overcome by insect detoxification enzymes. Additionally, phenolamides exert dual toxic effects targeting both insect midgut and nervous systems. Hydroxycinnamic acid amides disrupt the midgut peritrophic matrix and impede nutrient assimilation across multiple plant species (Gaquerel et al., 2017; Marti et al., 2013). Beyond gut damage, phenolamides suppress insect acetylcholinesterase activity and induce neurological dysfunction (Kaur et al., 2010; Liu Y et al., 2025). Given that the chemical structure of phenolamides differs markedly from BXs, the detoxification enzymes that S. frugiperda uses against BXs are unlikely to be effective against phenolamides. This observation is consistent with the classic “defense cocktail” hypothesis, where mixtures of diverse metabolites exert stronger effects on generalist herbivores than single compounds (Richards et al., 2016; López-Goldar et al., 2024). Our structure–activity analysis further shows that the methoxy group on the feruloyl moiety enhances bioactivity, consistent with reports that methoxylation increases membrane permeability or metabolic stability against insect detoxification enzymes (Onkokesung et al., 2012). Collectively, maize deploys a chemically diversified defense cocktail in which phenolamides are the major quantitative component, with BXs providing an additional layer of chemical diversity. Future studies should directly test this hypothesis by formulating artificial diets containing BXs, phenolamides, and their mixtures across a range of concentrations, followed by larval growth and survival assays. Such experiments will help resolve whether these two defensive metabolite classes act additively or synergistically across different concentration ranges, advancing our understanding of multi-layered plant anti-herbivore defenses.

We further explored the upstream signaling cascade regulating phenolamide defensive biosynthesis. Herbivory by FAW triggered pronounced accumulations of JA and JA-Ile levels, alongside transcriptional activation of core genes governing JA biosynthesis and signal transduction (Gaquerel et al., 2017). Notably, two maize MYC2 paralogs, MYC2a and MYC2b, were transcriptionally upregulated following larval infestation, with the induction magnitude of MYC2b far exceeding that of MYC2a. Collectively, these observations confirm that herbivore perception activates the JA signaling module in maize, and this hormonal cascade presumably governs the transcriptional induction of MYC2 transcription factors to orchestrate downstream phenolamide defense (Kaur et al., 2010; Ma et al., 2023; Li et al., 2024). Consistent with this, exogenous MeJA treatment rapidly induced *THT3* expression and phenolamide accumulation (**Supplementary Figure S3**). These data place the phenolamide pathway under the control of JA signaling module. Genetic analysis with *myc2* loss-of-function double mutants demonstrated MYC2 is essential for *THT* transcription and tyramine-derived phenolamide accumulation. The near-complete loss of phenolamide production in double mutants, coupled with its far stronger transcriptional induction upon FAW herbivory compared with *MYC2a*, prompted us to characterize the molecular function of MYC2b. MYC2 is universally recognized as the master regulator of JA-dependent anti-herbivore defenses (Kazan and Manners, 2013; Wasternack and Song, 2017). In maize, MYC2 is known to govern benzoxazinoid and volatile terpene biosynthesis (Ma et al., 2023; Li et al., 2024), yet direct control of phenolamide biosynthetic genes in grasses remained unreported. Yeast one-hybrid and dual-luciferase assays verified that MYC2b directly binds and activates the *THT3* promoter. This work is the first to document a direct MYC2b-;*THT3* regulatory module in grasses. Whether MYC2a also directly targets phenolamide biosynthetic genes remains to be experimentally determined. Our results extend maize JA signaling networks by defining a unique JA-MYC2b-*THT3*-phenolamide defensive branch parallel to MYC2-regulated BX and terpene pathways.

Our finding that N-feruloyltyramine impairs FAW larval performance aligns with a recent report of its insecticidal activity from *Humulus scandens* (Liu Y et al., 2025), but extends beyond this prior work by demonstrating that maize endogenously produces these compounds as an inducible defense, quantifying their absolute accumulation, revealing their quantitative dominance over BXs, and uncovering the direct MYC2-THT3 regulatory mechanism. From an applied perspective, the MYC2-phenolamide module offers a promising target for breeding or gene-editing strategies to enhance maize resistance, though potential trade-offs with growth or yield must be considered. Future research should address: (i) the relative field contribution of phenolamides versus BXs under multi-pest conditions; (ii) the molecular target(s) of these phenolamides in the insect gut; (iii) their potential synergy with BXs; and (iv) the conservation of this module across other cereals.Additional artificial-diet bioassays using basal pre-infestation concentrations of phenolamides would further verify whether only herbivore-induced metabolite levels are sufficient to constrain FAW performance. In conclusion, maize deploys a layered defense strategy—sustained, quantitatively dominant phenolamide accumulation superimposed on a rapid but transient BX burst—to ensure robust protection against FAW, advancing fundamental understanding of plant-herbivore interactions and opening avenues for sustainable crop protection.

## Data Availability

All data supporting the findings of this study are presented in the article and its supplementary materials. Metabolomics data are provided in **Supplementary Tables**.

## Supplementary material

Supplementary Table 1 Primer sequences used for qRT-PCR, Y1H, and dual-luciferase assays.

Supplementary Table 2 MRM parameters for LC-MS/MS analysis of phenolamides and benzoxazinoids.

Supplementary Table 3 List of differentially accumulated metabolites at 24 and 48 h (fold change, *P*-value).
