# Supplementary material for: MYC2b-regulated tyramine-derived phenolamides in maize defense against Spodoptera frugiperda
Source: Front Plant Sci. 2026 Oct 7;17:1945110. doi: 10.3389/fpls.2026.1945110 (PMC13683808; doi:10.3389/fpls.2026.1945110)
Supplement: Supplementary file 1 [file DataSheet1.docx]

**Supplementary Data**

**Supplemental Figure S1.** Quality control of metabolomics data. (A) Principal component analysis (PCA) score plot of QC samples showing tight clustering. Overlay of total ion current (TIC) chromatograms of QC samples at both negative ion mode (B) and positive ion mode (C), demonstrating high reproducibility.

Fig. S1A PCA scores of samples in different treatment groups and quality control samples

**Fig. S1B** MWXS-24-138-a_QC_MS_tic_overlap-N

**Fig. S1C** MWXS-24-138-a_QC_MS_tic_overlap-P

Notes: (A) PC1 denotes the first principal component, PC2 denotes the second principal component, and the percentage denotes the explanation rate of this principal component to the data set; Group is the grouping, the same component is denoted by the same color, and each point represents a component. Overlay plot of total ion chromatograms (TIC plots) from mass spectrometry detection of QC samples. The results show that the curves of total ion current for metabolite detection exhibit high overlap, with consistent retention times and peak intensities, indicating favorable signal stability when the same sample is detected by mass spectrometry at different times. The high stability of the instrument provides an important guarantee for the repeatability and reliability of the data, where N represents negative ion mode (B) and P represents positive ion mode (C).

**Supplemental Figure S2.** DIMBOA content in maize plants infested by FAW.

Notes: Absolute contents of DMBOA in maize leaves at 24 h and 48 h post infestation were analyzed by LC-MS. Data represent mean ± SD (n = 4). Statistical significance was analyzed using Tukey’s test in a one-way ANOVA (*P* < 0.05), with different letters indicating significant differences between treatments.

**Supplemental Figure S3.** Induction of *THT3* expression and phenolamide accumulation by MeJA.

Note: (A) Time‑course of Zm*THT3* expression after MeJA treatment (100 μM). (B) Accumulation of feruloyltyramine at 0, 24, and 48 h after MeJA treatment. Statistical significance was analyzed using Tukey’s test in a one-way ANOVA (*P* < 0.05), with different letters indicating significant differences between treatments.

**Supplemental Figure S4. Yeast single-hybrid and double-luciferase experiments to investigate the interaction between THTs and MYC2**

Note: (A) Cloning of the promoter fragment of the target gene; (B) PCR verification of bait strains constructed bacteriophages; (C) Detection of AbA resistance in bait yeast; (D) Transcription factor ZmMYC2 gene cloning and prey vector construction enzyme digestion verification.

**Supplemental Tables**

**Supplemental Table S1.** Primer sequences used for qRT‑PCR, Y1H, and dual‑luciferase assays.

**Supplemental Table S2.** MRM parameters for LC‑MS/MS analysis of phenolamides and benzoxazinoids.

**Supplemental Table S3.** List of differentially accumulated metabolites at 24 and 48 hpi (fold change, P‑value).
